# Midwifery

**Published:** 1825-01-01

**Authors:** 


### Midwifery.

"1. *Puerperal Fever.** Dr. Payne publishes this case of puerperal fever, with the view of establishing a different method of treating that disease, from that which, it appears, is still pursued by many practitioners, both in Edinburgh and London," viz. by blood-letting. The *object* of this paper is therefore highly important, and we esteem it our bounden duty, to enter as fully into the question as its importance demands.

Certainly, in order that the case given may illustrate and "*establish*" the proper treatment, it must be an unequivocal and undoubted instance of the disease in question. This is the first point to be established—then we may proceed to the merits of the remedy. Unhappily, however, for the reputation of the turpentine, we greatly fear that the disease which it cured, in this instance, was *not* the disease it was intended and supposed to cure.

The scope of our remarks will, hence, be mainly to point out the grounds on which we found our opinion of an error in the diagnosis. In doing this, we shall first endeavour to shew, that the symptom on which Dr. Payne depends for his diagnosis, is not pathognomonic of the disease. Secondly, that there are symptoms noticed as occnrring in this case, which denote a different disease; and, thirdly, that the mode of treatment was such as no practitioner would venture to confide in for the removal of an active internal inflammation, under any circumstances.

Dr. Payne *first* imagined the case of his patient *not* to be puerperal fever, *because* it was not "preceded by rigor;" then that, it *would* prove to be puerperal fever, or "peritoneal inflammation," because severe rigor at length occurred. Now it is scarcely possible that *both* these modes of thinking can be just, in their application to the *self-same* case, and on the *self-same day;* and we must either conclude that inflammation did already exist, although rigor had not yet occured, or that, even after the rigor, there was not inflammation; for it must be observed, that the case was not one of insidious character, but one of the most acute and urgent kind.

Thus, admitting for a moment that the case was puerperal fever, it is plain that, contrary to Dr. Payne's experience and axiom, it was NOT *preceded by rigor.* We now proceed to shew that the symptoms of the disease were such, from the very commencement, as denote intestinal irritation, and not inflammation. But, as we must be as brief as possible, we shall merely copy the account of the symptoms given in the commencement of the case, "there were," then, "much head-ache, urgent thirst, intolerance of light, and great disturbance produced by the slightest noise." Now these are precisely the symptoms which arise from intestinal irritation,

* Case of Puerperal Fever, successfully treated by Oil of Turpentine. By Henry Payne, M.D. *Ed. Med. Journal, for July* 1824, *p.* 53.

and which are, to say the least, by no means essential to inflammation, if, indeed, they were *ever* observed in a case of *pure* inflammation.

Then, in the third place, the case was *severe*, and yet ceased *without the use of the lancet.* Now, has any one ever known inflammation of the peritoneum, in its severer form, trusted to any remedy but the lancet? has any other violent internal inflammation ever been trusted to any other remedy than the lancet? and would any one, in the present state of our knowledge, ever have the hardihood to trust puerperal or any other *active internal inflammation* to any other remedy? We answer, undoubtingly and unhesitatingly, No.—If such disease were to yield, the fact must be learnt by *accident*, not by *design*; for we imagine that no one will dare to try the experiment.

Dr. Payne's case is, however, a very instructive one, and would have been truly valuable, had it been given with the view, not of establishing a different mode of the treatment of puerperal fever, but of illustrating the effects of intestinal irritation; and whilst we earnestly beg of our junior readers, not to be misled by it into the neglect of the lancet in actual inflammation, we recommend it to their attentive perusal, as a good and fair example of that other disease. And we would especially notice an occurrence on the 5th day. Dr. Payne observes, "on my return from a ride into the country, I learnt that a messenger had called, requesting my attendance as soon as possible. I understood there had been considerable pain of the head, and some tendency to delirium." On the preceding evening, the patient had indulged too freely in conversation on a distressing subject; and the previous night had not been very good. Dr. Payne proceeds, "as the abdominal pain was entirely removed, some practitioners, on seeing approach the tendency to delirium, might suppose there was going to take place a metastasis to the brain." Dr. Payne was not, however, deceived in this matter: and although we cannot approve of his mode of reasoning, or of his sweeping dogma relative to the catholicon power of his favourite remedy, we wish to do him the justice of observing, that we believe he was quite correct in his distinction of the *symptoms* and *appearance* of affection of the head from real phrenitis;—"but being in the habit," he adds, "of seeing my puerperal patients recover under the use of turpentine, I felt confident that the unpleasant symptoms would quickly be removed by the application of cold to the forehead." The whole case is interesting, when viewed as one of intestinal irritation, or perhaps, indeed, as an illustration of the utility of the turpentine in this particular kind of puerperal disease, and we think it altogether very well and accurately given.

---

2. *Puerperal Fever.** We are at a loss to imagine the author's object in the publication of this case, which we notice for two reasons,—*first*, to point out the vague and unsatisfactory manner in which cases are apt to

---

* Case by H. Sanders, Surgeon. Med. and Phys. Journal, No. 307, for September, p. 214

be given to the public, now-a-days;—for instance—"Tuesday, had passed urine freely, and without pain; after-pains trifling, lochia natural. Wednesday, the abdomen has become tense and swollen. The pain previously complained of, has returned with increased violence; can only lie on the right side. *Neither the pulse nor tongue indicated the presence of active inflammation.* Salines, with Dover's powder, were prescribed, with a saline cathartic. Thursday, pain somewhat increased on pressure, and *the symptoms more inflammatory*," &c. &c. Now on the Wednesday, the symptoms were *not* inflammatory at all; on the Thursday, they were *more* inflammatory. But we do not intend merely to object to the *verbal* inaccuracy of this narration: for we should be contented had we only substantial *facts*, however faulty the phraseology; but we should have been glad to know *why* there were *no* inflammatory symptoms on Wednesday, and more on Thursday; or, rather, why the author concluded it so to be. It ought to have been his object in *publishing* his case, to have satisfied *us*; as it is destitute of all *detail* of symptoms, and, consequently of all evidence for the author's opinion, it is not, as a published case, worth one farthing. But, *secondly*, the case is really useful, as exemplifying one particular fact, which we have very frequently noticed, viz. that *there may be severe rigor in puerperal cases, without any serious consequence.* Thus—"Tuesday. Was sent for in great haste, supposing my patient was *in articulo mortis.* On arriving, *she had just recovered from a severe rigor:* though excessively low and dejected, her countenance had assumed a more favorable aspect; she had had some refreshing sleep; her pulse about 120, and more regular; her thirst had diminished; tongue cleaner, and less dry; could bear pressure better, and had been lying on the right side for three hours. The blister had risen well, and the pain in the thigh had nearly abated. I continued the diaphoretic saline, applied a second blister to the sacrum, and allowed her a more generous diet.—Wednesday. Her bowels costive; had not acted since Tuesday forenoon. Ordered a common cathartic enema.—Thursday. Much better in general, though the pain had a little increased. The bowels had twice freely acted, from the injection. I applied six leeches to the groins, and continued the saline. From this time, my patient became gradually convalescent." Now it is by no means our intention to inculcate the doctrine, that rigor does not require the strictest attention and subsequent watching; for this it certainly *does* require; but we wish to impress upon our readers the fact, for such we assuredly know it to be, that rigor, in the course of a puerperal affection, does not *always* indicate the occurrence of inflammation, or suppuration; but that it is sometimes induced by other causes, and especially, we believe, by that state of the bowels, and of the system at large, which precedes, attends, or follows the action of purgative medicine; still, however, let us *watch* carefully after rigor,—but with *two* objects in view, viz. to detect early the *first* or *second* of these events, and to discriminate between them and the *third*.

